# Groups for belonging: a parallel cluster randomized controlled trial of a group-based loneliness intervention for people attending treatment for alcohol and other drug use

**DOI:** 10.1186/s12889-025-24369-6

**Published:** 2025-08-27

**Authors:** Isabella Ingram, Genevieve Dingle, Briony Larance, Amanda L. Baker, Frank P. Deane, Laura D. Robinson, Alison K. Beck, Lucy Leigh, Ava Kontogiannis, Elle P. Coleman, Tayla J. Degan, Bethany Nixon, Robert Stirling, Suzie Hudson, Leanne Hides, Scott Drummond, Danielle Breeze, Adrian Webber, Kevin Street, Anthea Bill, Emily Matthews, Peter J. Kelly

**Affiliations:** 1 School of Psychology, University of Wollongong, Wollongong, NSW Australia; 2 Centre for Alcohol Policy Research, La Trobe University, Melbourne, VIC Australia; 3 School of Psychology, University of Queensland, Brisbane, QLD Australia; 4 National Drug and Alcohol Research Centre, University of New South Wales, Sydney, NSW Australia; 5 Data Sciences Unit, Hunter Medical Research Institute, University of Newcastle, Newcastle, NSW Australia; 6 Network of Alcohol and other Drugs Agencies, Sydney, NSW Australia; 7 Drug Policy Modelling Program, University of New South Wales, Sydney, NSW Australia; 8 Centre for Alcohol and Other Drugs, New South Wales Ministry of Health, Sydney, NSW Australia; 9 Victorian Alcohol and Drug Association, Melbourne, VIC Australia; 10 Kedesh Rehabilitation Services, Berkeley, NSW Australia; 11 Alcohol & Other Drugs and Mental Health, Uniting Care, Melbourne, VIC Australia; 12 Health Research Economics, Hunter Medical Research Institute, University of Newcastle, Newcastle, NSW Australia

**Keywords:** Loneliness, Belonging, Intervention, Substance use, Experience, Satisfaction, Randomized controlled trial

## Abstract

**Background:**

People accessing alcohol and other drug (AOD) treatment experience high rates of loneliness, which is a strong driver of substance use. Groups for Belonging is a 6-session group-based intervention that combines elements of the social identity informed intervention Groups for Health with psychoeducation and strategies to help participants manage loneliness and overcome cognitive barriers to social connection. The project will examine the effectiveness and cost-effectiveness of delivering *Groups for Belonging* within routine care offered by AOD treatment providers in Australia.

**Methods:**

Participants will be adults attending residential or community-based AOD treatment provided by either public sector or non-government treatment providers across New South Wales (NSW), Victoria (VIC), Queensland (QLD) and the Australian Capital Territory (ACT) in Australia. The study will be conducted as a parallel cluster randomized controlled trial (RCT), involving 26 clusters (13 treatment, 13 control, *N* = 520), with randomization occurring at the service level. Depending on randomization, participants will either complete treatment as usual (i.e., AOD treatment provided by participating services) or Groups for Belonging and treatment as usual.

**Discussion:**

This study aims to determine the effectiveness of a loneliness intervention, *Groups for Belonging*, for people accessing residential and community-based treatment for AOD use. If the intervention is effective and cost-effective, implementation efforts will focus on embedding the intervention into routine care in AOD treatment services across metropolitan and regional areas in Australia. This has significant potential to decrease loneliness and its associated effects on health and wellbeing for people accessing AOD treatment.

**Trial registration:**

Australia and New Zealand Clinical Trials Registry, CTRN12625000132448 (https://www.anzctr.org.au/ACTRN12625000132448.aspx). Registered on 6 February 2025.

**Supplementary Information:**

The online version contains supplementary material available at 10.1186/s12889-025-24369-6.

## Background

Loneliness is a public health priority. It is estimated that up to 25% of the population experience loneliness, with estimated rates rising worldwide, and impacting people of all ages [1]. Although widespread, we are not ‘all in the same boat’ when it comes to loneliness. Those who have poorly-met financial needs are 2.8 times more likely to be lonely (51%) than those with well-met financial needs (27%) [2]. In Australia, 39% of those living in the most disadvantaged communities report being lonely, compared to 28% of those in the least disadvantaged neighborhoods [2]. Often falling in the lower socioeconomic band, people accessing treatment for alcohol and other drug (AOD) use are seven times more likely to experience loneliness and perceived their loneliness to be five times more serious than people in the general population [3]. Loneliness is a significant predictor of poor physical and mental health, and increased morbidity and mortality rates [4]. People accessing treatment for AOD use experience poor health and an increased propensity to chronic diseases [5]. The high rates of loneliness observed across people accessing AOD treatment are likely to contribute to further health disparities in this population.

A review of 41 studies examining loneliness in people with problematic AOD use found loneliness was associated with poor physical and mental health, as well as an increased frequency, severity and duration of substance use amongst this population [6]. The review also identified that targeted loneliness interventions that meet the specific social needs of this population are lacking. Only one intervention had been trialed with people accessing AOD treatment and it found the intervention had no effect in reducing loneliness relative to control [7]. The study was conducted four decades ago, and the intervention was broad in scope, highlighting a need for more up to date, targeted interventions. People who are accessing AOD treatment experience unique social challenges as substance use is typically a key part of the social network when they enter treatment, but once they reduce, or are no longer using substances, their social needs change. No longer identifying as strongly with substance using social groups when addressing AOD use can trigger feelings of isolation, making treatment engagement a particularly vulnerable time for these individuals [8, 9].

There are key cognitions and behaviors that contribute to, and maintain, loneliness for people who use AOD to a problematic extent. A qualitative study with people accessing AOD treatment found that mistrust, perceived lack of support from others, low self-worth, fear of negative evaluation, poor quality and authenticity in relationships, and unhelpful interpersonal behaviors were both key contributors to, and consequences of, loneliness [10]. Additionally, participants identified that their substance use contributed to feelings of loneliness and that these feelings led to engaging in, or re-engaging in, problematic AOD use [10]. In this study, participants indicated that support groups, identifying and pursuing authentic relationships and engaging in activities that provide a sense of purpose would likely help appease feelings of loneliness. In response to this need and the study’s findings, a novel loneliness intervention, *Groups for Belonging*, was developed (see [11]).

*Groups for Belonging* is a 6-session (2-hours per session) group-based intervention that is based on a social identity approach to health as well as cognitive theories of loneliness. The social identity approach suggests that the groups and communities that we belong to help to shape our identity and sense of belonging, and that loneliness is a result of a loss or lack of group memberships and identities. *Groups for Belonging* adapts some elements from the social identity informed intervention, Groups for Health [12], an evidence-based intervention for socially isolated adults that has been examined using both proof of concept and RCT study designs. Groups for Health has demonstrated reductions in loneliness and related distress amongst university students and adults accessing mental health services in Australia [12, 13]. *Groups for Belonging* uses some elements from Groups 4 Health such as psychoeducation about the influence of social connections on health, and a personalised mapping of social groups.

In addition, cognitive theories suggest that the way we think of ourselves and others in relationships guides the way we will interact and behave in relationships, which in turn will determine how connected we feel to others [14]. *Groups for Belonging* aligns with cognitive theories of loneliness by helping participants to identify and address cognitive barriers to social connection such as stigma, social anxiety and difficulty trusting others (see [10]). A feasibility trial (*N* = 41) of *Groups for Belonging* in residential AOD treatment services revealed high levels of satisfaction with the program, with 89% reporting the highest possible satisfaction rating [15]. While the study was underpowered to detect significant effects of the intervention on loneliness and related outcomes, it pointed to a need for an adequately powered RCT to determine the effectiveness of *Groups for Belonging* for people accessing treatment for AOD use.

## Objectives

This study will examine the effectiveness and cost-effectiveness of delivering *Groups for Belonging* within routine care offered by AOD treatment service providers in Australia. It is hypothesized that compared to treatment as usual, *Groups for Belonging* will be associated with greater reductions in loneliness (primary outcome variable). Secondary outcomes will examine cost effectiveness, the impact of group participation on substance use, social group membership, physical health, mental health, and wellbeing.

## Methods

### Design

**Fig. 1:**
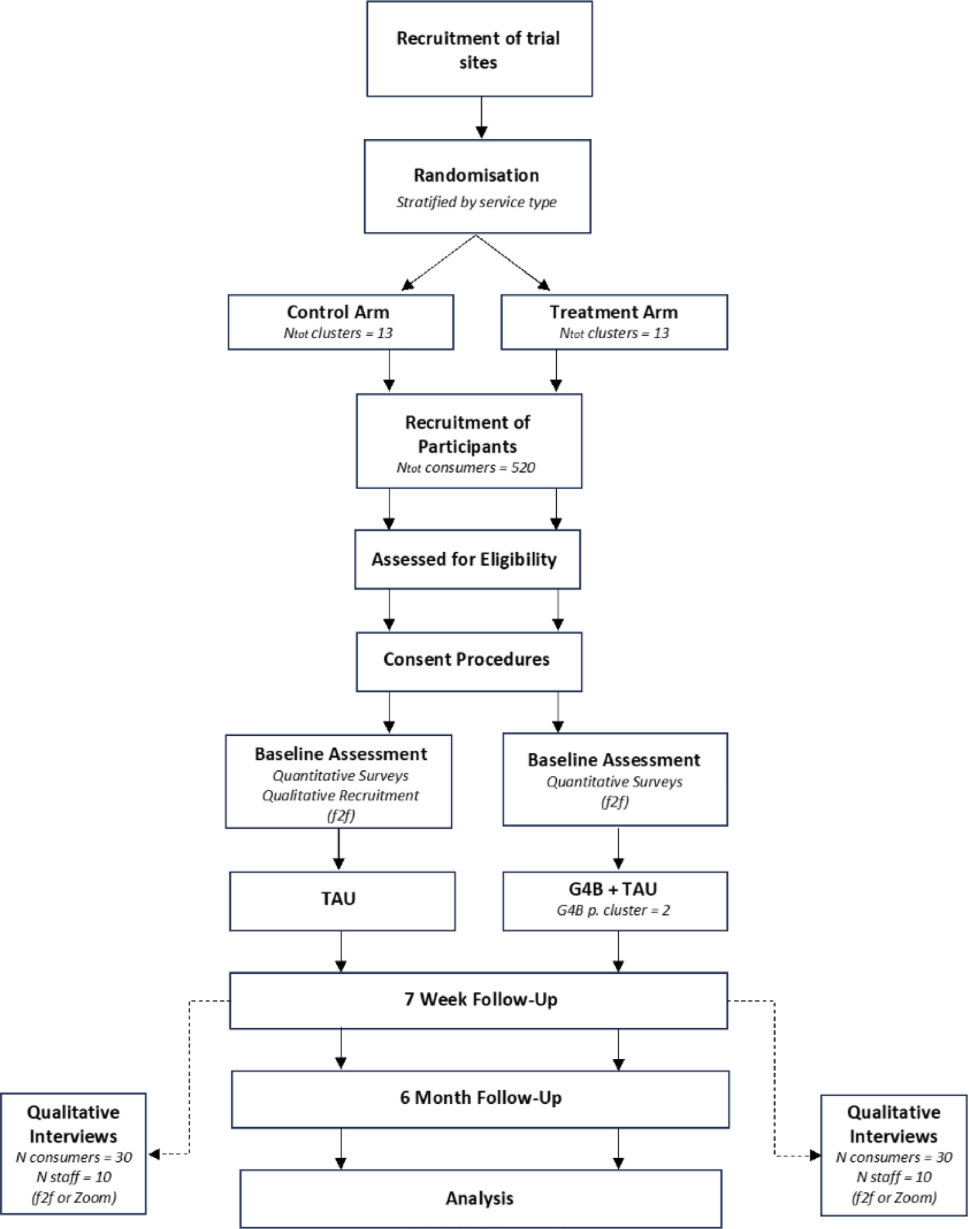
Flow chart of study procedure

The study will be conducted as a parallel cluster-RCT, involving 26 clusters (13 treatment, 13 control), with randomization occurring at the service level. Depending on randomization, participants will either complete *treatment as usual* (TAU; i.e., treatment for AOD use provided by participating services) or *Groups for Belonging* and treatment as usual. Assessments will be conducted at baseline, 7-weeks (post intervention), and 31-weeks (6-months post-intervention). Assessment officers, blind to treatment condition, will collect the primary and secondary outcome measures at each of these timepoints. The study design was developed in consultation with a lived experience advisor, as well as multiple Consumer Advisory Groups and these consultations will occur throughout the duration of the project. The protocol follows Standard Protocol Items: Recommendations for Interventional Trials (SPIRIT) guidelines (see Supplementary File 1 SPIRIT checklist). Figure 1 describes the study flow.

### Setting

Participants will be attending residential or community-based programs provided by either public sector or non-government AOD treatment providers across the Australian states of New South Wales (NSW), Victoria (VIC), Queensland (QLD) and the Australian Capital Territory (ACT). The programs offered by each of these services will be diverse, however, all services will offer AOD treatment programs that are accessed by participants for a minimum of 5-weeks. Recruitment of the trial sites will be facilitated by the peak bodies for non-government services across those states, and through consultation with state provided public health services. To promote the generalizability of findings, sites will be located across metropolitan and regional areas of Australia.

### Participants

A total of 520 adults will be recruited to participate in the study and will be involved in either the treatment (Groups for Belonging, *n* = 260) or control (treatment as usual, *n* = 260) condition depending on which condition the site that they are accessing has been randomly allocated to.

#### Inclusion and exclusion criteria

Participants will be required to: (i) be an adult (aged 18 + years) currently accessing treatment for AOD use (i.e., not for gambling); (ii) have a current interest in improving social connections; (iii) intend to access treatment for at least 5-weeks; and (iv) have access to a mobile telephone to complete follow-up assessments. Exclusion criteria will be kept to a minimum to promote the generalizability of the findings. Participants will be excluded from the study if they are currently experiencing acute suicidality or unstable mental health symptoms as determined by themselves, their case workers, or the research clinicians.

### Randomization

The randomization procedures will be managed by an independent statistician based at the Hunter Medical Research Institute (HMRI). Block randomization will be used to randomize clusters to each arm in a 1:1 ratio. Randomization will be stratified by service type (residential or community) to ensure balance of service types across the study arms. Treatment as usual will be used as the comparator condition to ascertain whether the intervention (*Groups for Belonging*) is effective above and beyond the standard services offered within the AOD treatment services setting.

### Conditions

#### Treatment condition (Groups for belonging and TAU)

**Fig. 2:**
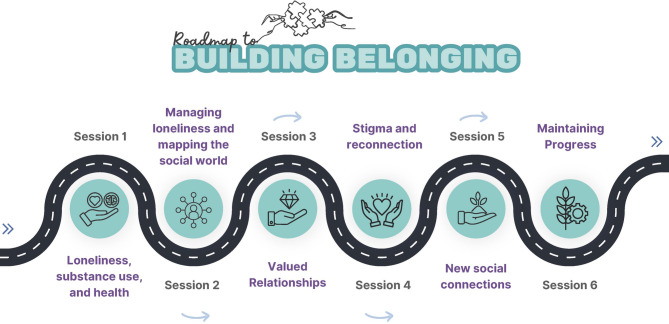
Session outline of groups for belonging

Participants allocated to the treatment condition will complete treatment as usual in addition to completing Groups for Belonging [17]. Groups for Belonging involves 6 × 2-hour sessions (*n* = approximately 10 participants per group) delivered over a 4 to 6-week period, depending on the length of the program offered by respective treatment sites (i.e., up to 2 sessions can be delivered per week where necessary). *Groups for Belonging* is based on cognitive theories of loneliness, which posit that unhelpful cognitions act as barriers to connection [14], and a social identity approach which suggests that identification with social groups enhances connection and belonging [18, 19]. The program aims to help participants understand the connection between loneliness and substance use, identify relationships that might be barriers or facilitators to their AOD treatment goals, to identify and manage the cognitions (e.g., stigma, mistrust, fear of negative evaluation, fear of showing feelings) that act as barriers to connection, and to develop goals and strategies that help to promote positive social connections. Figure 2 outlines the key aspects of the *Groups for Belonging* program.

#### Control condition (TAU)

Participants allocated to the control condition will continue with treatment as usual (the AOD treatment program offered by the community/residential service that they are attending).

### Recruitment

Study information (e.g., flyers, posters, and participant information sheets) will be available at each treatment site. Research clinicians will also visit the sites to meet with potential participants to discuss the study. Alternatively, staff of the treatment services will provide the research officers with the names and contact details of potentially interested consumers. At each site, potential participants will be asked to complete a brief expression of interest form. On this form, the eligibility criteria will be outlined. If participants are interested in the project, the research clinician will contact the consumer via telephone, or in person where practicable, and provide more information about the study, review the eligibility criteria, and complete the informed consent procedures.

### Participant retention

Participant retention will be facilitated by obtaining detailed contact information and multiple secondary contacts. Research clinicians will attend the treatment services to facilitate follow-up data collection and promote retention in the study. For each assessment completed, participants will be reimbursed with an AUD$50 cash, gift card or direct debit into their nominated bank account, according to participant preference. Text message reminders will also be sent to participants 48 h prior to their scheduled follow-up assessments.

### Trial monitoring

#### Intervention delivery–training and supervision

Research clinicians will be employed to facilitate the *Groups for Belonging* intervention onsite at each of the AOD treatment services involved in the project. These clinicians will complete formal training led by the developers of the intervention, who are experienced clinical psychologists (II and GD). The intervention will be co-facilitated by a nominated staff member of each AOD treatment service. The clinical psychologists will also provide weekly supervision and coaching to research clinicians and treatment service staff delivering the intervention.

#### Treatment fidelity

A clinical psychologist and experienced researcher (AKB) will oversee the training and supervision of fidelity assessors. *Groups for Belonging* sessions will be audio recorded, and the trained Fidelity Assessor will rate a random allocation of 20% of treatment sessions (stratified by session and study site) for adherence and competence. Instruments used to measure fidelity and competence were developed and selected using steps outlined by Walton, Spector [20]. A fidelity checklist was developed using the Behavior Change Technique Ontology [21] to accurately capture the content of the *Groups for Belonging* sessions. The manualized group intervention check [22] was selected to capture clinician competence using a 30-item instrument.

#### Adverse events

Auditing of trial conduct will be undertaken by the trial coordinator (EC). Adverse events will be recorded by clinical researchers and assessment officers and managed by the trial coordinator (EC) throughout the duration of the trial according to specific steps approved by the overseeing ethics committee (Royal Prince Alfred Hospital HREC; 2024/ETH00914). Necessary steps will be taken to ensure participants are linked in with appropriate services and support.

### Data collection

Demographic characteristics including date of birth, age, postcode, gender, identification as Aboriginal and/or Torres Strait islander, marital status, sexual orientation, dependents/children, living situation, accommodation, education level, employment, job status, history of treatment for AOD use, history of mental and/or physical health conditions, and use of medications will be collected at baseline. All primary and secondary outcomes will be collected at baseline, 7-weeks (i.e., post intervention) and 31-weeks (i.e., 6-months post intervention) and entered directly into Research Electronic Data Capture (REDCap) platform using a laptop (see Table 1). Baseline assessments will be completed either via telephone or, where possible, onsite at the AOD treatment services. Blinded assessment officers will collect the primary and secondary outcome measures via telephone at all timepoints, while research clinicians will collect all other measures at baseline as a means of building rapport with participants. The blinded assessment officers will be trained in the administration of the outcome measures and will receive weekly supervision with one of the senior team members (TD). The assessments will be reviewed weekly for completeness to minimize missing data and improve accuracy. As part of the baseline assessment procedure, contact details for the participant and a nominated alternate contact will be collected to facilitate completion of the follow-up assessment procedures.

### Follow-up

Follow-up will be conducted via telephone at 7-weeks after baseline and again at 6-months post-intervention. To ensure blind assessment of outcome measures, the primary and secondary dependent measures will be collected by an assessment officer who is naïve to allocation and study design. At the beginning of each phone call, participants will be reminded that they should not reveal their condition to the assessment officer. If participants disclose their condition, an alternative assessment officer will complete the assessment. Assessments will take approximately 30 min to complete. Multiple methods will be used to stay in contact with participants (e.g. telephone, email, SMS, social media). Alternate contact details provided by the participant will be used if there is difficulty reaching the participant. Table 1 reflects a schedule of assessments, including the primary and secondary outcomes collected at each time point.

**Table 1:** Schedule of assessments

Domain	Data		Assessment Interval			
		Pre-Trial	Baseline	Intervention period	7-w	6-m
Recruitment	Eligibility	x ^c^				
Demographics						
	Date of Birth		x^b^			
	Age		x^b^			
	Postcode		X^b^		x^a^	x^a^
	Gender		X^b^			
	Aboriginal and/or Torres Strait Islander		x^b^			
	Marital status		x^b^			
	Sexual orientation		x^b^			
	Dependents/children		x^b^			
	Living arrangements		x^b^			
	Accommodation		x^b^			
	Education		x^b^			
	Employment		x^b^			x^a^
	Job status		x^b^			
	Previous treatment episode		x^b^			
	Mental health diagnoses		x^b^			
	Physical health conditions		x^b^			
	Medications		x^b^			x^a^
Substance use						
	TLFB (i.e. days of use)^2^		x^a^		x^a^	x^a^
	Treatment goals		x^b^		x^a^	x^a^
	Cravings (DAQ-6)^2^		x^a^		x^a^	x^a^
Loneliness						
	Single item		x^b^			
	Drinking Motivations		x^b^			
	UCLA (Version 3)^1^		x^a^		x^a^	x^a^
Social variables						
	Multiple group membership^2^		x^a^		x^a^	x^a^
	SEMSI		x^b^		x^a^	x^a^
	Use/recovery identity		x^b^		x^a^	x^a^
	Support for treatment goals		x^b^		x^a^	x^a^
Barriers to connecting						
	Self-stigma		x^b^		x^a^	x^a^
	Mini-SPIN		x^b^		x^a^	x^a^
	Young Schema Questionnaire (mistrust, emotional inhibition, defectiveness/shame)		x^b^		x^a^	x^a^
Wellbeing						
	Psychological health (K10)^2^		x^a^		x^a^	x^a^
	Suicidality (CSSRS-S)		x^a^		x ^a^	x ^a^
	Quality of life (AQoL-8D)^2^		x^a^			x^a^
Experience of Care						
	YES				x^a^	
	PREMAT				x^a^	
Service use						
	CSRI^2^		x^a^			x^a^
	Travel^2^		x^b^	x^c^	x^a^	
G4B experience (treatment arm only)						
	Group Climate Questionnaire				x^c^	
	8-item program evaluation				x^c^	

^a^Assessment done via telephone with blinded Assessment Officer or via electronic link. ^b^Assessment done with research clinician. ^c^Completed independently by participant. ^1^Primary Outcome. ^2^Secondary outcomes. *TLFB* Timeline Follow Back, *DAQ-6* Desires for Alcohol Questionnaire, *UCLA* University of California Los Angeles Loneliness Scale, *SEMSI* Self-Efficacy in Managing Social Identities, *Mini-SPIN* Mini Social Phobia Inventory, *K10* Kessler 10, *AQoL-8D* Assessment of Quality of Life – 8 Dimensions, *YES Survey* Your Experience of Service Survey, *PREMAT* Patient Reported Experience Measure for Addiction Treatment, *CSRI* Client Services Receipt Inventory, *G4B* Groups for Belonging

### Primary outcome

#### Loneliness

The 20-item UCLA Loneliness Scale (Version 3) [23] will be used. This is the most widely used and robust loneliness measure. It has been recommended for people experiencing mental health conditions, over the abbreviated versions of this scale [24]. This scale has been designed to measure an individual’s subjective feelings of loneliness. An example item is “How often do you feel that no one really knows you well”. Response options include: 1 - never, 2 - rarely, 3 - sometimes or 4 - often. A total score is computed, and higher scores indicate greater loneliness [25].

### Secondary outcomes

#### Days of substance use

The Timeline Follow back (TLFB) method will be used to obtain information about duration of abstinence and days of substance use. The TLFB is the “most psychometrically sound” self-report measure of substance use [26, 27]. Participants are presented with a calendar and are asked to provide retrospective data on their days of substance use over a 28-day period prior to their treatment entry [26].

#### Cravings

The Desires for Alcohol and Drugs Questionnaire (DAQ-6) will be used to assess cravings for substances. Participants are prompted to select their drug of preference and then asked to rate their agreement towards 6- items on a seven-point Likert scale from 1- Strongly agree, to 7- Strongly disagree [28]. An example item is “I would do almost anything to have a drink/use drugs right now”. A total score is computed with higher values indicating greater cravings.

#### Group membership

The Multiple Group Memberships Scale [29] (4-items adapted from the Exeter Identity Transition Scale) will be used to determine identification with social groups. Items include questions about participants’ perception of their group membership. An example item is “I belong to lots of different groups”. Response options range from 1- Do not agree at all, to 7 – Agree Completely. Scores are averaged across the items, with a higher average score indicating greater group membership.

#### Psychological distress

The Kessler Psychological Distress Scale (K10; [30]) will be used to assess psychological distress. An example item is “In the past 4 weeks, about how often did you feel hopeless”. Each item is rated on a five-point Likert scale ranging from 1 - None of the time, to 5 - All of the time. Responses are summed to yield a score of between 10 and 50, with higher scores signaling greater levels of distress.

#### Quality of life years adjusted (economic analysis)

The Assessment of Quality of Life (AQoL-8D) [31] will be used to assess health related quality of life. This instrument will determine the cost-effectiveness of the Groups for Belonging intervention. The AQol-8D consists of 35-items and has two overarching domains, including physical and psychosocial quality of life. An example item is “How well do you communicate with others”. Higher scores on these domains indicate higher health related Quality of Life.

#### Service use (economic analysis)

A Client Service Receipt inventory (CSRI; [32]) will be used to ascertain the types and amount of service use participants have engaged in at baseline and at the 6-month timepoint. The CSRI includes items about AOD service use, health services and hospital service use, contact with the criminal justice system, and mode of travel to these services.

### Other variables

#### Loneliness

A single item “I often feel very lonely” will be used to report prevalence of loneliness. Response options for this item are: 1 – Strongly disagree to 7 – Strongly agree. This item is taken from the Household Income and Labour Dynamics in Australia (HILDA) Survey [33] and will allow for the prevalence of loneliness to be compared to the general Australian population.

A single item from the Drinking Motivations Questionnaire (DMQ-R; [34]) will be used to determine how frequently substance use was motivated by the following reasons: “so you won’t feel left out” (item 20). This item was adapted and added to read “so you won’t feel lonely”. Response options are: 1 - Almost Never/Never to 5 - Almost always/Always.

#### Treatment goals

A single item will be used to determine treatment goals: “Thinking about your primary substance(s) of concern, which of the following items best fits your goals?” Responses include: Total abstinence, never use again; Total abstinence but realize a slip is possible; Occasional use when urges strongly felt; Temporary abstinence; Controlled use; No goal.

#### Self-efficacy in managing identities

The Self-efficacy in Managing Social Identities Scale (SEMSI; [35]) will be used to examine participants’ self-efficacy in managing their identities. The SEMSI is 5-items, with response options rated on a 5-point Likert scale ranging from 1 – Strongly disagree, to 5 – Strongly agree. An example of an item on the SEMSI is “I am confident that I can make meaningful connections with members of the groups that I belong to”. Items are summed together for an overall score, with higher values indicating greater self-efficacy.

#### Suicidality

The Columbia Suicide Severity Risk Scale - Screener (CSSRS-S; [36]) will be used to assess suicidal ideation. Suicidal ideation severity is rated on a five-point ordinal scale with 1 - yes, or 0 - no responses. Points on this scale tap into the following themes: 1 - wish to be dead, 2 - non-specific active suicidal thoughts, 3 - suicidal thoughts with methods, 4 - suicidal intent, and 5 - suicidal intent with plan. A score of 3 or greater on the CSSRS–S indicates potential risk of suicide at any assessment time point.

#### Support for treatment goals

A single item was adapted from the Assessment of Recovery Capital [37] to ascertain whether participants have support to pursue their treatment goals. This item reads: “I have a network of people I can rely on to support my treatment goals”. Response options for this item are: 1 - Yes or 2 - No.

#### User/recovery identity

Three items will be used to determine participants identity in relation to their substance use. The items include “I identify with other drug users/drinkers”, “I identify with others in recovery from addiction”, and “I identify with other non-users/non-drinkers”. Responses are on a 5-point Likert scale ranging from 1 - Strongly disagree to 5 – Strongly agree, with higher scores reflecting higher identification with that group.

#### Barriers to connecting

Self-Stigma will be assessed using the Internalized Stigma of Mental Illness Inventory (ISMI-9; [38]). The ISMI-9 contains 9 items. Responses are on a Likert scale ranging from 1 – Strongly disagree to 4 – Strongly agree. The current measure was adapted by the research team in consultation with a consumer advisory group. The term ‘mental illness’ was changed to ‘problematic drug and/or alcohol use’ to adequately reflect stigma specific to the study population. An example item is “I feel out of place because of my problematic drug and/or alcohol use”. Scores from the 9 items are summed together for a total score, and higher scores reflect greater self-stigma.

Fear of Negative Evaluation will be assessed using a brief version of the Social Phobia Inventory, the Mini-SPIN [39]. The Mini-SPIN consists of three items with response options ranging from 0 – Not at all, to 4 – Extremely, which are summed together for a total score. An example of an item is “Do you avoid activities in which you are the center of attention”. Higher scores reflect greater fear of negative evaluation.

To measure theorized barriers to connecting with others (see [10]) subscales of the Young Schema Questionnaire (YSQ-SF; [40]) will be used that relate to: Mistrust, Emotional Inhibition, and Defectiveness/Shame. Each subscale consists of five items, and response options range from 1 – Completely untrue, to 6 – Describes me perfectly. An example of an item from the mistrust subscale includes “I feel that people will take advantage of me”. A total score for each subscale will be computed and higher scores indicate greater theorized barriers to connecting with others.

#### Patient experiences of care

The Patient-Reported Experience Measure for Addiction Treatment (PReMAT) [41] was designed in partnership with people accessing residential AOD treatment services and comprises 33 items that reflect aspects of care most important to people attending these services. An example item is “My day is structured here”. Each item is responded to on a 5-point Likert scale from 1 - strongly disagree to 5 - strongly agree, with total scores ranging from 31 to 155. Higher scores indicate greater levels of positive experience.

To evaluate *participant experience [with Groups for Belonging and TAU]* we will use the Your Experience of Service (YES) Survey [42]. The YES Survey is Australia’s national measure of consumer experience in mental health services. This national questionnaire was developed in partnership with mental health consumers and comprises 26 items designed to gather information from consumers about their experience of care. An example item is “Staff showed respect for how you were feeling”. The 22 items related to ‘experience’ and 4 items related to ‘outcomes’ yield response options ranging from 1 - Never to 5 - Always or 1 - Poor to 5 - Excellent. Scores for two scales (Experience Scale and Outcome Scale) will be summed to provide two summary scores.

### Measures specific to the treatment condition

Group experience will be collected at Week 7 using an 8-item scale developed by our team to examine the experience of completing Groups for Belonging. An example of these items include: “I was able to have a say and share my experiences”. Participants can respond “yes” or “no” to each item.

To evaluate group cohesion over the 6 sessions of *Groups for Belonging*, the Group Climate Questionnaire – Short form (GCQ-S; [43]) will be used at Week 7. The 12 items of the GCQ-S are divided into three subscales measuring *engagement*,* conflict*, and *avoidance*. An example item is “The members liked and cared about each other”. Items across subscales are rated on a 7-point Likert scale from 0 - Not at all, to 6 - Extremely. Scale scores are determined by calculating the average of relevant items.

### Analyses

#### Power analysis

Sample size calculations were performed using the STATA package ‘clustersampsi’ [44]. The control mean loneliness score was assumed to be 49 (*SD* = 10), and intervention mean of 45 (*SD* = 10), based on findings reported across similar samples [45, 46] and indicators of significant and clinically meaningful change across more diverse samples [47]. Intra Cluster Correlation was assumed conservatively to be 0.05. It was calculated that 13 clusters per arm, with 20 participants recruited per cluster, would have 81% power and 5% type 1 error rate to detect a difference in loneliness at 6 months of 4 points. A retention rate of 70% was assumed based on the research team’s track record of achieving excellent follow-up rates across similar samples [48, 49].

#### Data analysis

Statistical analyses will be managed by independent statisticians. Loneliness scores will be analyzed using linear regression in a mixed modelling framework. Fixed effects will include terms for intervention, time point (7 weeks or 6 months), an interaction between intervention and time point, baseline loneliness score, and strata (community-based vs. residential), and random effects for cluster and participant will be included to account for clustering by site, and repeated measurements on participants. The estimated mean difference in loneliness scores between the arms at the primary (6 months) and secondary (7 weeks) time points will be reported, along with 95% confidence intervals, and p-values, as summary measures of the intervention effectiveness.

The analysis set for the primary outcome will be Intention to Treat, however, sensitivity analyses using the Per Protocol analysis set will also be performed. In the case of missing data, multiple imputation methods will be utilized to ensure the analysis set is Intention to Treat. Analysis of secondary outcomes at 7 weeks and 6 months will also utilize linear mixed models (if assumptions of normally distributed residuals and constant variance are met) or generalized linear mixed models otherwise. Fixed effects will include those listed above for the primary outcome variable. Effect estimates for the difference between arms, 95% confidence intervals, and p-values, will be reported for each outcome at each follow-up time point. A sub-group analysis will be performed estimating the difference in change in loneliness from baseline among those participants who reported high loneliness scores at baseline. A secondary sub-group analysis comparing the difference in loneliness scores at 7 weeks and 6 months for residential participants compared to community-based participants will also be performed.

### Economic analysis plan

A trial-based economic evaluation will be conducted from a health system perspective based on Intention to Treat. Resource use data associated with participation in the intervention, and treatment as usual will be prospectively identified, measured, and valued. Outcomes included in the analysis will be aligned to the primary trial outcome – incremental change in loneliness as well as change in health-related quality of life, measured using the AQoL-8D [31] to be transformed into incremental Quality Adjusted Life Years. Incremental cost-effectiveness ratios (ICERs) will be calculated by dividing the between-group difference in costs by the between-group difference in effects. Uncertainty intervals around the ICERs will be estimated using non-parametric bootstrapping techniques and graphically presented on cost-effectiveness planes. Acceptability curves and net monetary benefit will also be calculated. Sensitivity analysis will test for variability in parameters such as unit costs and event likelihood. The analysis will incorporate results from the CSRI, to capture patterns of downstream service use and associated costs. Given the expected longer-term impact of improving mental wellbeing among this population, a lifetime model will be specified and populated to understand the longer-term value of *Groups for Belonging*.

### Qualitative study

In the treatment arm, participants who attend *Groups for Belonging* (anticipated *N* = 30, depending on point of saturation), and service staff who co-facilitate *Groups for Belonging* (i.e., research staff and clinicians employed at sites, *N* = 10), will be interviewed about their experience of the program and asked to reflect on the key aspects of *Groups for Belonging* (treatment processes and content) that they perceive contribute to belonging. The interviews will be conducted following the completion of the *Groups for Belonging* program. Qualitative interview data in the treatment arm will be generated to understand barriers and facilitators surrounding the implementation of *Groups for Belonging*, and perceived mechanisms by which the program may work to reduce loneliness.

Demographic and descriptive information will be synthesized using descriptive statistics. Qualitative zoom interviews will be audio recorded (with prior consent from participants) and sent for transcription by a professional transcriber working under a confidentiality agreement. Data coding and analyses will be guided by iterative categorization. Iterative categorization is compatible with common analytical approaches (e.g. thematic analysis) and offers a clear, standardized guide for coding which allows for replication and validity in qualitative data analysis [50].

### Experience sub-study

During the consultation with service providers in the development of the research protocol, it was identified that services providers in the control condition would value the collection of satisfaction and experience measures to help inform continuous service improvement following the completion of the formal RCT. Subsequently, a mixed methods sub study was developed that is focused on understanding the experience of treatment for people accessing treatment as usual. Satisfaction and experience measures have been embedded in the protocol (i.e. YES survey and PREMAT) to provide a mechanism to examine the relationship between satisfaction and experience with outcomes. Likewise, in the control arm, consumers (anticipated *N* = 30) and service staff members (anticipated *N* = 10) will be interviewed about their experience of care in accessing (consumers) or delivering (staff) treatment. The interviews will be conducted either in person (where possible) or online following the 7-week assessment timepoint (consumers) or following consent procedures (staff). Qualitative interview data in the control arm will be generated to examine factors that consumers and service staff perceive as effective or ineffective. This will cover aspects of the facility, program elements, and interactions with both staff and other consumers at the service they are using.

## Discussion

Loneliness is almost *seven times* more prevalent for people accessing AOD treatment than the general population [3]. Social isolation has been found to contribute to deaths related to substance use [51], and yet, no interventions targeting loneliness and social isolation have so far been evaluated in this population. This study aims to determine the effectiveness and cost-effectiveness of a loneliness intervention, *Groups for Belonging*, for people accessing residential and community-based AOD treatment services across Australia. *Groups for Belonging* has been designed to meet the specific social needs of people with substance use disorders, such that it targets key cognitions that contribute to, and maintain, feelings of loneliness. This includes barriers to connecting due to stigma, fear of negative evaluation, difficulty trusting others, and emotional inhibition. A feasibility study of *Groups for Belonging* was conducted in residential AOD treatment services, and found the intervention was regarded positively amongst consumers and feasible for delivery across these services [15]. Groups for Belonging is a novel, consumer informed, intervention which has potential to be scalable. If found to be effective and cost effective for delivery across both community-based and residential services in the current study, the intervention will be disseminated across the AOD treatment sector.

### Strengths and limitations

The study poses various strengths, particularly its focus on the “real world”, by aiming to examine the effectiveness of a loneliness intervention when implemented in routine care across AOD treatment services. Since the research closely aligns with real clinical practice, it will provide reliable information about the effectiveness of the group-based loneliness intervention when embedded into routine care. The study also aims to improve the generalizability of the findings by minimizing exclusion criteria. Additionally, having assessment officers who are blind to treatment conditions collect primary and secondary outcomes helps reduce bias and enhance the validity of the findings. Finally, the study has been designed to accurately monitor aspects of intervention fidelity (e.g., training and support of clinicians, monitoring delivery of the intervention) to maximize the confidence in findings of the trial.

Potential challenges may include the prospect of a low recruitment rate and/or high participant attrition. To maximize recruitment, a desire to improve social connections has been specified as an eligibility criterion, rather than loneliness, since there may be some reluctance to disclose feelings of loneliness throughout the recruitment process (due to social stigma associated with loneliness, e.g., [16]) and there is an established high prevalence of loneliness across similar samples [3]. Sub-group statistical analyses will help to account for this as a potential limitation. Additionally, the power analyses have adopted a conservative approach to ensure sufficient power. The study team has demonstrated a strong track record of successfully recruiting both services and participants for similar studies. To further safeguard these potential limitations, the research team includes both advisors with lived experience and representatives from key non-government AOD treatment providers to support recruitment and site retention. Additionally, to facilitate participant retention and minimize drop out, multiple strategies will be used, including remuneration for participation, obtaining detailed contact information, and identifying multiple secondary contacts.

## Conclusions

Loneliness is highly prevalent and problematic amongst people accessing AOD treatment. The *Groups for Belonging* intervention has been specifically designed to meet and support the needs of people accessing AOD treatment. It is hypothesized that compared to treatment as usual, *Groups for Belonging* will be associated with greater reductions in loneliness, and improvement in other health and wellbeing outcomes. If found to be effective, *Groups for Belonging* can be implemented as part of routine care in treatment services across both metropolitan and regional/rural areas in Australia.

## Supplementary Information

Below is the link to the electronic supplementary material.

Supplementary Material 1

## Data Availability

The datasets used and/or analyzed during the current study are available from the corresponding author on reasonable request.

## Abbreviations

AOD: Alcohol and other drugs
TAU: Treatment as usual
SPIRIT: Standard protocol items: recommendations for interventional trials
HMRI: Hunter Medical Research Institute
REDCap: Research electronic data capture
TLFB: The Timeline Follow back
ICERs: Incremental cost-effectiveness ratios
